# Correction to 5 papers to correct the issue number

**DOI:** 10.1093/ecco-jcc/jjag093

**Published:** 2026-07-21

This is a correction to the following 5 papers:

https://doi.org/10.1093/ecco-jcc/jjaf080

https://doi.org/10.1093/ecco-jcc/jjaf069

https://doi.org/10.1093/ecco-jcc/jjaf070

https://doi.org/10.1093/ecco-jcc/jjaf075

https://doi.org/10.1093/ecco-jcc/jjae192

In the originally published versions of these manuscripts, the PDF files contained erroneous issue information.

These errors have been corrected so that DOI: jjaf080 reads as assigned to “Volume 19, Issue 6” and the other four papers on the above list read as assigned to “Volume 19, Issue 5”.

